# Custom-Made Implant Fabrication for Chin Augmentation Using Piled-Up Expanded Polytetrafluoroethylene Sheets: An Innovative Surgical Technique and Literature Review

**DOI:** 10.1007/s00266-024-03918-1

**Published:** 2024-03-18

**Authors:** Jing-Song Guo, Kwan Lok Benjamin Ng, Su-Shin Lee, Ya-Wei Lai, Yi-Chia Wu

**Affiliations:** 1 Department of Surgery, Kaohsiung Medical University Hospital, Kaohsiung Medical University, Kaohsiung, Taiwan; 2 Division of Plastic Surgery, Department of Surgery, Kaohsiung Medical University Hospital, Kaohsiung Medical University, No.100, Tzyou 1st Road, Kaohsiung, 807 Taiwan; 3 The Bishop Clinic, 6F, No.6, Heping E Road, Da’an District, Taipei, Taiwan; 4 Faculty of Medicine, College of Medicine, Kaohsiung Medical University, Kaohsiung City, Taiwan; 5 Regenerative medicine and cell therapy research centre, Kaohsiung Medical University, Kaohsiung, Taiwan

**Keywords:** Chin augmentation, e-PTFE, Cosmetic surgery, Innovation

## Abstract

**Background:**

Alloplastic chin augmentation is the most common esthetic surgical treatment to reshape the chin. However, factory-made chin implants are typically standardized rather than custom-made and have potential to cause complications. Although the fabrication of custom-made implants by using computer-assisted planning and 3D-printing technology has become widespread, the process has several disadvantages, including long preoperative prosthesis preparation times, high costs, and unsuitability for patients with asymmetric chins or those who undergo combined mandibuloplasty before implant placement. The present study developed an innovative chin augmentation technique involving stacked expanded polytetrafluoroethylene (e-PTFE) sheets that is suitable for most patients and has minimal side effects.

**Materials and Methods:**

A retrospective review of a single surgeon’s experience was performed over a 2 year period for patients who underwent a procedure involving piled-up e-PTFE sheets for alloplastic chin augmentation. This study analyzed the outcomes, complications (temporary nerve numbness, wound infection, hematoma formation, and implant displacement), and patient satisfaction during follow-up.

**Results:**

Between January 2018 and December 2020, 38 patients underwent the procedure involving piled-up e-PTFE sheets for alloplastic chin augmentation. Six patients (15.8%) experienced nerve-related temporary numbness, and one (2.6%) experienced wound infection. None had developed major complications such as implant displacement or wound infection at follow-up. Moreover, the patients demonstrated a high level of satisfaction with the surgical results.

**Conclusion:**

Piled-up e-PTFE sheets can be used to produce custom-fit porous polyethylene chin implants that result in minimal complications and a very high satisfaction rate.

**Level of Evidence IV:**

This journal requires that authors assign a level of evidence to each article. For a full description of these Evidence-Based Medicine ratings, please refer to the Table of Contents or the online Instructions to Authors www.springer.com/00266.

## Introduction

A short, retruded, or deviated chin causes the face to appear asymmetric and unbalanced, which may negatively affect self-esteem and self-confidence. Chin augmentation is a popular facial plastic surgery procedure in which the chin contour is reshaped to achieve a balanced, esthetically pleasing appearance. To achieve the optimal surgical outcome, a surgeon must perform a complete preoperative facial evaluation, thoroughly understand the patient’s anatomy, and have well-honed surgical skills. Clear communication between the surgeon and patient is vital to achieving satisfactory outcomes. Two types of chin augmentation are typically performed: (1) sliding genioplasty, which requires manipulation of the chin bones and relocation of the anterior portion of the mandibular bone to draw the chin out, and (2) alloplastic chin augmentation, in which an implant is employed to reshape the chin. Both methods have been well studied, and each has advantages and disadvantages. However, alloplastic chin augmentation is typically the preferred procedure for reshaping the chin.

An ideal implant material is inert, noncarcinogenic, and nonreactive. The inner portion of the implant should be flexible enough to fit the underlying facial structures, whereas the outer portion should blend with the mandibular bone to provide a smooth chin line without a gradient. Silicone, chimeras, polyurethane foams, artificial bone grafts, and expanded polytetrafluoroethylene (e-PTFE) are widely used materials for chin augmentation. However, silicone implants have a smooth surface that may cause bone erosion, capsular contracture, and a high incidence of implant displacement [1]. By contrast, polyurethane foams are spongy and have a low rate of capsular contracture, whereas chimeras and e-PTFEs are composed of polyester fibers with pores. The porous structure of chimeras and e-PTFEs works with soft tissue ingrowth to produce secure fixation and sufficient tensile strength to resist distortion. Among the aforementioned materials, e-PTFE is the most ideal for implants, and it has few disadvantages [2–4]. However, although e-PTFE is flexible and can easily be shaped, precisely sculpting the inner and outer portions of the implant is challenging.

Computer-assisted planning and 3D-printing-assisted modeling for chin augmentation have been proposed by several studies as methods for fabricating custom implants that fit precisely to the mandible and blend with this bone to produce a smooth chin line [5]. However, in addition to possible errors in the 3D-printing process, this surgical technique has the disadvantage of potentially not being suitable in many cases because patients usually receive combined osteotomy, mandibuloplasty, or mandible reduction surgery during chin augmentation, rendering precise preoperative sculpting of such implants difficult.

In this paper, we describe an innovative surgical technique involving stacked e-PTFE sheets being used to fabricate a custom-made implant for chin augmentation with few side effects. This article presents our experience in implementing this modified chin augmentation procedure.

## Methods and Materials

This study consists of a retrospective review of medical records from the authors’ clinic. All patients provided written informed consent to receive the surgical procedures and for their case data and images to be published for academic purposes.

We reviewed the medical records of patients with short, retruded, or deviated chins who underwent chin augmentation surgery involving piled-up e-PTFE sheets between January 2018 and December 2020.

Patients were eligible for the procedure if they (1) had a short, retruded, or asymmetric chin, (2) had prior chin surgery with an unnatural and unsatisfactory outcome, or (3) were dissatisfied with the appearance of their chin.

### Surgical Technique

The operation was performed with each patient under intravenous sedation and analgesia.

**Fig. 1:**
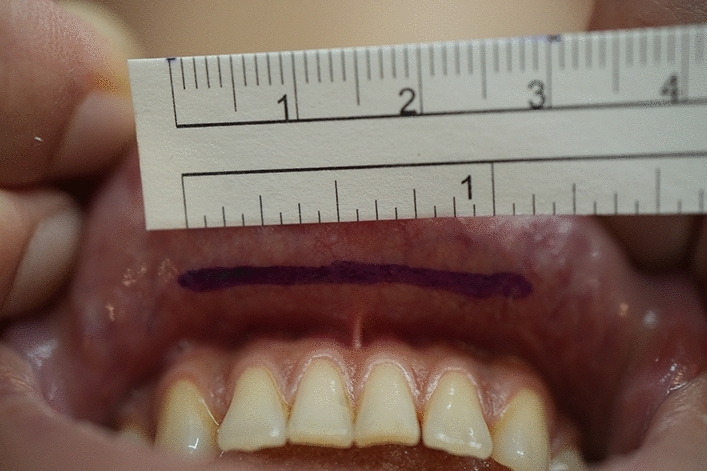
The incision line was marked with methylthioninium chloride

**Fig. 2:**
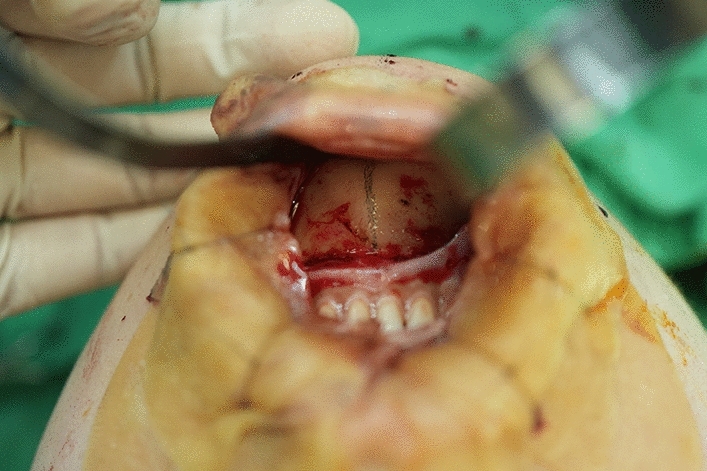
Dissection through the mentalis muscle along to the periosteum was performed. Subperiosteal dissection was carried until the mental protuberance and the genial tubercles were fully exposed

**Fig. 3:**
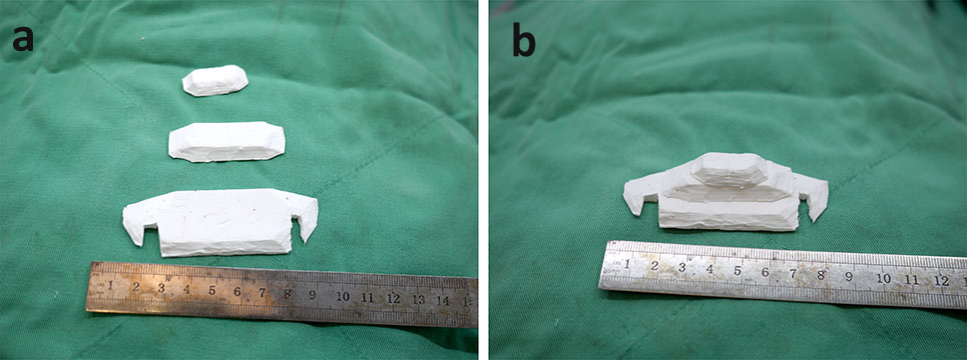
**a** e-PTFE sheets were designed and sculpted, **b** e-PTFE sheets were piled up for implant fabrication

**Fig. 4:**
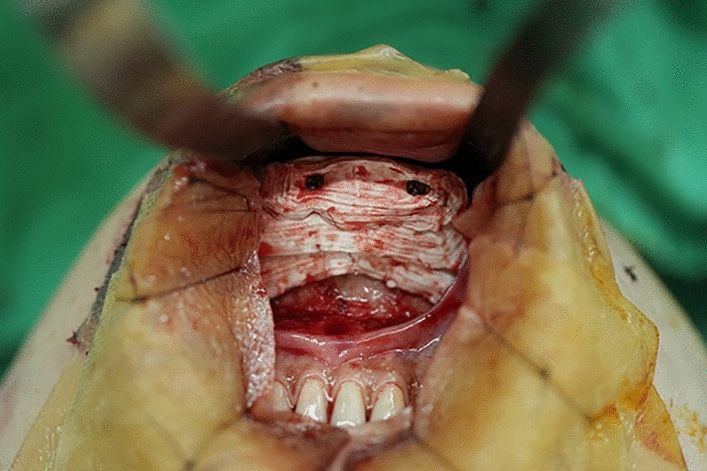
The implant was inserted to the dissected space and fixed with two screws

**Fig. 5:**
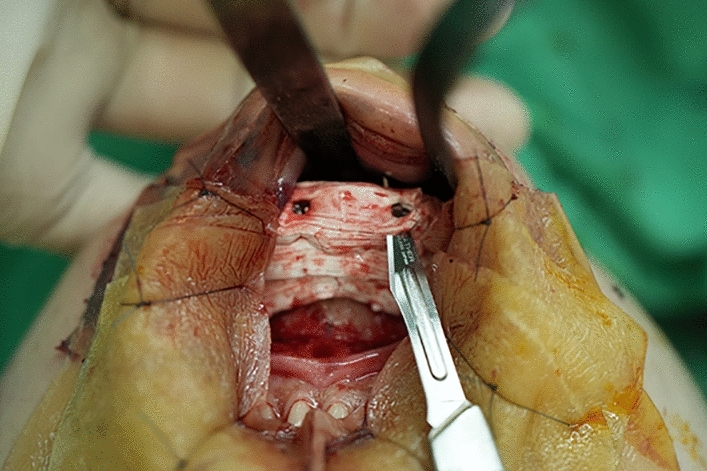
Excessive areas of the implant were carved away using a size 15 scalpel until a patient-specific custom shape was obtained

**Fig. 6:**
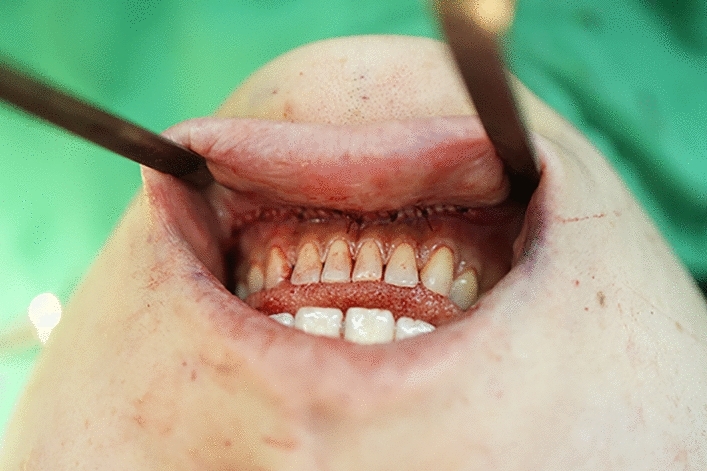
The muscle layer and mucosa layer were closed with 4-0 PDS and 4-0 chromic sutures, respectively

An intra-oral approach was used, with the surgeon marking the incision line with methylthioninium chloride (Fig. 1). A mixture of 1% lidocaine and 1:100,000 epinephrine was infiltrated into the dissection area. A transverse incision, approximately 3 cm long, was made 5 mm above the buccal sulcus in the midline of the vestibular mucosa. Dissection was performed through the mentalis muscle along the periosteum. Subperiosteal dissection was performed using a periosteal elevator until the mental protuberance, and the genial tubercles were fully exposed (Fig. 2). Precautions were taken to avoid injury to the mental neurovascular bundles. e-PTFE sheets were stacked until the desired shape—which varied from patient to patient—was created (Fig. 3). For each implant, when more than three layers of e-PTFE sheets were stacked, the base layer of the implant had to be widened to create a natural chin shape. Two holes, as presented in Fig. 3, were created to prevent compression of the mental nerve. After successful hemostasis and irrigation of the operation field with antibiotic solution, the stacked e-PTFE sheets were soaked with antibiotic solution (1 gm of cefazolin in 250 mL normal saline solution) and inserted into the dissected space. Two titanium screws were used to precisely and securely fix each implant to the mandible (Fig. 4). The compressibility of the e-PTFE sheets enabled the inner surfaces of the implants to be custom-shaped and automatically fitted to the mandible. The outer contours of the implants were then refined using a scalpel. The implants were further sculpted by removing excess material until a final, patient-specific shape was obtained with smooth bilateral chin lines (Fig. 5). The large Gore-Tex pieces that were carved out were removed using forceps, and for each patient, the surgical site was irrigated with normal saline to ensure no Gore-Tex debris remained. Before wound closure, the dissection space was again irrigated with an antibiotic solution. The muscle layer and mucosa layer were closed with 4-0 PDS and 4-0 chromic sutures, respectively, to create a water-tight barrier between the oral cavity and the dissected space (Fig. 6). A chin dressing with mild compression was applied for 2 weeks postoperatively to reduce swelling and prevent hematoma formation. Postoperative care included a soft diet, chin ice-packing, and proper oral hygiene. Additionally, oral antibiotics were administered for 7 days to prevent infection.

### Explantation Technique if Necessary

If explantation was required, the soft tissue was required to be separated from the prosthesis at the proper plane, with the proper plane line lying on the surface of the prosthesis. Electrocautery was required to dissect the soft tissue until the prosthesis was exposed. After the prosthesis was exposed, dissection was made along the prosthesis surface to free the implant. Because the prosthesis was stacked in layers, removing the prothesis layer by layer was a simple process.

In cases of infection, explantation was assisted by the accumulation of exudates within the dissection plane that naturally separate the prosthesis from the chin.

### Follow-Up and Outcome Measures

Patients were closely monitored after surgery. Outcomes were smoothness of the bilateral chin line, chin appearance at 1 month following the operation, complication rates, and patient satisfaction. Patient satisfaction was assessed at 12 months by using a questionnaire survey and a 10-point scale. (A score of 10 represents the most positive experience, whereas a score of 1 represents the most negative experience.) All patients were examined by the same surgeon who performed the surgery.

### Statistical Analysis

Continuous variables are expressed as means ± standard deviations, and categorical variables are expressed as counts (percentages). Statistical analyses were performed using IBM SPSS, version 22, for Windows (IBM, Armonk, NY, USA).

## Results

### Patient Characteristics

The study population comprised 32 women and 6 men with a mean age of 33.5 years and with an age range of 22–48 years. The patient characteristics are presented in Table 1. Of the 38 patients, 28 (73.7%) received primary chin augmentation, and 10 (26.3%) had prior chin surgery at other clinics and requested reoperation due to dissatisfaction with previous cosmetic outcomes.

**Table 1:** Patient demographics and case characteristics

Sex	
Female	32 (84.2%)
Male	6 (15.8%)
Number of surgeries	
Primary chin augmentation	28 (73.7%)
Revision chin augmentation	10 (26.3%)
Complications	
Nerve temporary numbness	6 (15.8%)
Wound infection	1 (2.6%)
Hematoma formation	0 (0%)
Implant displacement	0 (0%)
Mean follow-up time	37 months
Mean 10 points satisfaction scale	8.1/10

### Outcomes

**Fig. 7:**
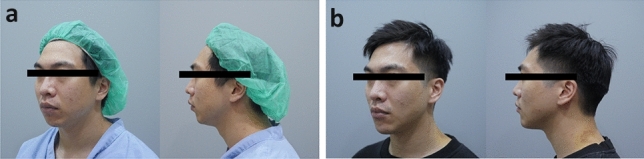
Preoperative and postoperative images from representative cases

**Fig. 8:**
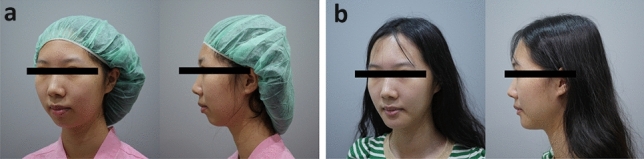
Preoperative and postoperative images from representative cases

All surgeries were performed without immediate major complications, including nerve injury, postoperative infection, or hematoma formation. All patients were closely monitored, and they returned to the clinic for follow-up at 1, 3, 6, and 12 months after the surgery. The mean follow-up time was 37 months. During follow-up, no implant displacement or hematoma was identified, although infection was noted in one patient, for whom implant removal was performed, and 500 mg of oral amoxicillin was prescribed. After the infection was brought under control, revision chin augmentation was arranged without event or noticeable side effects. The surgeon and the patients evaluated the postoperative outcomes during the 12 months of follow-up. All 36 patients were satisfied with the cosmetic outcomes, with the average satisfaction rating being 8.1 points. Preoperative and postoperative images from representative cases are presented in Figs. 7 and 8.

## Discussion

The primary goal of facial esthetic surgery is to create an appearance of symmetrical, harmonious proportions [6]. In cases where the chin is short, retruded, or asymmetric, recontouring may be considered. Alloplastic chin augmentation is the most widely used method for adjusting the lower chin contour. Various chin implants made of a variety of materials have been used to augment the mental area [7]. However, no single implant is suitable for all patients and most are not custom-made to suit the unique contours of different patients’ chins.

**Fig. 9:**
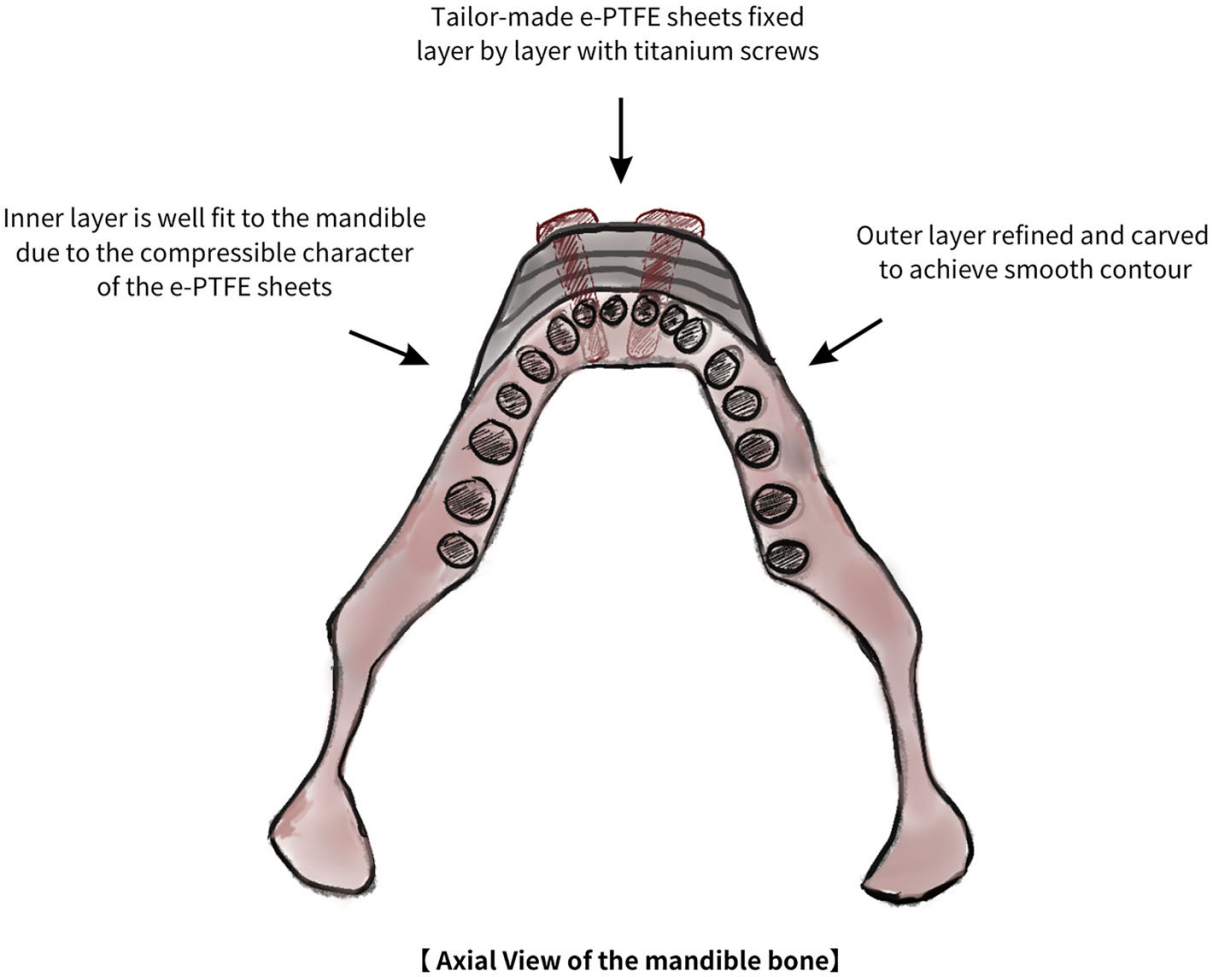
Illustration of the piled-up e-PTFE sheets implant

An ideal chin implant must meet two criteria. First, the implant’s inner portion must be precisely fitted to the mandible, which prevents implant displacement, excessive bulkiness, or an unnatural appearance after surgery. Second, the outer portion of the implant must blend well with the mandible to ensure that the bilateral chin lines are smooth and natural. Factory-made implants may be composed of various materials, such as silicone, chimeras, or e-PTFEs. These factory-made implants share several disadvantages: (1) They have a standardized size and shape and are not custom-fit; (2) the inner portion of the implant frequently poorly fits the mandibular structures; (3) patients may face complications such as implant displacement, unnatural postoperative facial contours, or bone erosion [8]; (4) the outer portion of the implant can be carved and sculpted intra-operatively but is not always cut precisely enough to create a smooth chin line; (5) an unsightly palpable postoperative border commonly occurs; (6), factory-made implants are typically symmetric along the left–right axis, making them difficult to employ in patients with deviated or asymmetric chins; (7) they often lead to complications such as malposition, infection, and paresthesia [8]; and (8) 1.8–2.6% asymmetry typically results when factory-made implants are used for chin augmentation, with a general revision rate ranging from 0.85 to 1.7%. By contrast, the procedure employed in the present study has several advantages: (1) the e-PTFE sheets are inserted in the dissection area layer by layer to create a 3D shape that is custom-fitted to each patient; (2) the inner portion of the implant precisely fits the mandible because of the high compressibility of the e-PTFE sheets (Fig. 9); (3) the porous sheets allow for soft tissue ingrowth, providing early stabilization that, when combined with screw fixation, enables the implant to match the facial bones with low risk of displacement [9]; (4) the e-PTFE sheets can easily be sculpted and shaped with scissors, scalpels, or other readily available equipment; (5) skilled intra-operative implant sculpturing using e-PTFE sheets creates a smooth outer chin contour that is naturally attractive (Fig. 9); and (6) the stacked e-PTFE technique is suitable for patients with asymmetric chins because sheets can be layered over asymmetrical areas to reduce asymmetry.

Donets et al. reported the fabrication of a custom-made implant using computer-assisted planning and 3D-printing technology [10]. In that study, a CT scan of the patient’s skull was performed preoperatively, and subsequently, a 3D model of the mandible was created to assess the bone’s shape and size. After, the new projection and desired shape of the chin were discussed with the patient, and a chin implant was custom-made, with an inner portion precisely fitted to the mandibular structures and an outer portion that blended with the mandibular body. Because the implant was custom-made for the patient, the satisfaction rate was high. Chang et al. fabricated a similar custom-made implant and reported a high satisfaction rate [11]. Although such methods of chin augmentation are often successful, they also have several drawbacks. First, 3D printing may not be as accurate as is often supposed. Several studies have investigated the accuracy of models fabricated using 3D printing. For example, Yeung et al. conducted an in vitro study comparing the accuracy of 3D models created using different 3D printers. Their results revealed that although most 3D-printing systems were accurate enough for clinical use, substantial differences in accuracy were observed between different 3D-printing systems [12]. Additionally, Kim et al. evaluated the accuracy of 3D-printed implants and reached a similar conclusion to that of Yeung et al. [13], reporting a mean absolute difference in 3D-printing systems ranging from 0.06 to 1.93 mm. Second, most patients who undergo chin augmentation require simultaneous osteotomy, mandibuloplasty, or mandible reduction surgery. The fit of custom-made implants created using computer-assisted planning and 3D printing may be compromised when these additional surgical procedures are performed during chin augmentation. However, e-PTFE sheets are naturally sufficiently flexible and compressible to fit any mandibular structure. Moreover, fabricating the piled-up e-PTFE sheets in the present study does not require complex preoperative examinations or X-rays.

An additional challenge often encountered with fabricating implants is maintaining the shape and volume of the implant after the operation. In one study, Jung et al. used high-resolution ultrasonography [14] to evaluate the thickness of e-PTFE implants in patients receiving rhinoplasty and observed no substantial changes in implant thickness over time. Additionally, Godin et al. reported on their experience with 324 cases of chin augmentation completed using e-PTFE implants [15], observing no resorption or displacement of implants within the study period. These findings suggest that no substantial volume or shape changes occur following alloplastic chin surgery involving e-PTFEs.

Another advantage of the e-PTFE surgical procedure is its brevity. Custom-made chin augmentation involving stacked e-PTFE sheets has an average surgical time of less than 1 h from incision to closure.

Intra-oral e-PTFE sheets offer many advantages to the Asian populations served by the authors of the present study. First, such populations have a high prevalence of hypertrophic scars, with these scars being particularly common in patients of Chinese descent, as indicated by Tsang et al. suggests [16]. In addition, Best et al. concluded in a systemic review that the complication rate (including the rate of infections) when the intra-oral approach adopted in the present study is employed is comparable to that when the extra-oral approach is employed [17].

Although stacked e-PTFE sheets offer many advantages, they also have disadvantages. First, no objective criteria have been established to quantify surgical outcomes, which are currently evaluated only through preoperative and postoperative photograph comparisons and questionnaire surveys. Nevertheless, beauty is subjective, and patient satisfaction is the primary determinant of success in esthetic surgery. Second, substantial training is required to learn the procedure, and numerous skills are required to perform it. The difficulty of the procedure lies in the preparation and intra-operative sculpturing of the implant to ensure a smooth jawline postoperation. To achieve an optimal outcome, preoperative discussions with patients, intra-operation fabrication of the implant, implant contour refinement, and a keen eye for esthetics are crucial. Third, e-PTFE carries a high risk of local inflammatory reactions that may cause erythema and edema at the wound site that lead to infection. To minimize the infection rate, securely closing the wound is essential. After the mentalis muscle is closed, the wound must be protected from salivary leakage. Aseptic procedures and proper irrigation of the operation room are critical in this process. In the present study, no major infections were reported. Finally, e-PTFE sheets are expensive and may not be affordable for all patients. The authors of the present study concede that the operation is somewhat expensive; however, the accuracy and flexibility of the technique and the low rate of complications may well be worth the cost, as evidenced by the high satisfaction ratings of the patients in the present study.

## Conclusions

The results of this study illustrate the applicability of e-PTFE sheets for fabricating a custom-made implant for chin augmentation. The study demonstrates the possibility of chin augmentation using a tailor-made implant with a precise fit between the implant and the mandible to produce a smooth and natural contour. Additionally, the placement of a custom-fit implant is associated with a lower risk of nerve injury and implant migration. The satisfaction rate of the patients in the present study approached 100%. This surgical technique can be strongly recommended to a wide range of patients from diverse backgrounds.

## Abbreviations

e-PTFE: Expanded polytetrafluoroethylene
CT: Computed tomography
